# A Rare Case of Symptomatic Double Optic Disc Pit without Maculopathy

**DOI:** 10.1155/2016/2560568

**Published:** 2016-08-28

**Authors:** Zaria Ali, Mohammad Abdul-Nabi

**Affiliations:** ^1^Manchester Royal Eye Hospital, Oxford Road, Manchester M13 9WL, UK; ^2^East Lancashire Teaching Hospitals, Haslingden Road, Blackburn BB2 3HH, UK

## Abstract

Optic disc pits are an uncommon congenital abnormality. Patients remain asymptomatic unless they develop maculopathy. We present a rare case of a double optic disc pit of which only three others have been reported worldwide. A 51-year-old gentleman presented with blurred vision. Fundoscopy revealed a right double optic disc pit. Though he was symptomatic there was no evidence of maculopathy. OCT of macula and disc was otherwise unremarkable. Visual field demonstrated a paracentral defect. Although optic disc pits are rare they are still an important clinical entity. Prompt identification and treatment of complications are required to prevent a poor visual prognosis.

## 1. Introduction

Optic disc pits are a rare congenital abnormality [1, 2]. In 85–90% of cases they occur singly and unilaterally, with a small percentage occurring bilaterally [2]. Patients remain asymptomatic unless they develop maculopathy [3]. We present a rare case of a 51-year-old male presenting with a unilateral double optic disc pit of which only three others have been reported worldwide. Furthermore, his presentation is unusual due to presence of visual disturbance without evidence of maculopathy.

## 2. Case Report

A 51-year-old gentleman was referred with a six-month history of blurred vision in the right eye and a suspicious right optic disc. He did not complain of any other ocular symptoms and did not have any other past medical history.

**Figure 1:**
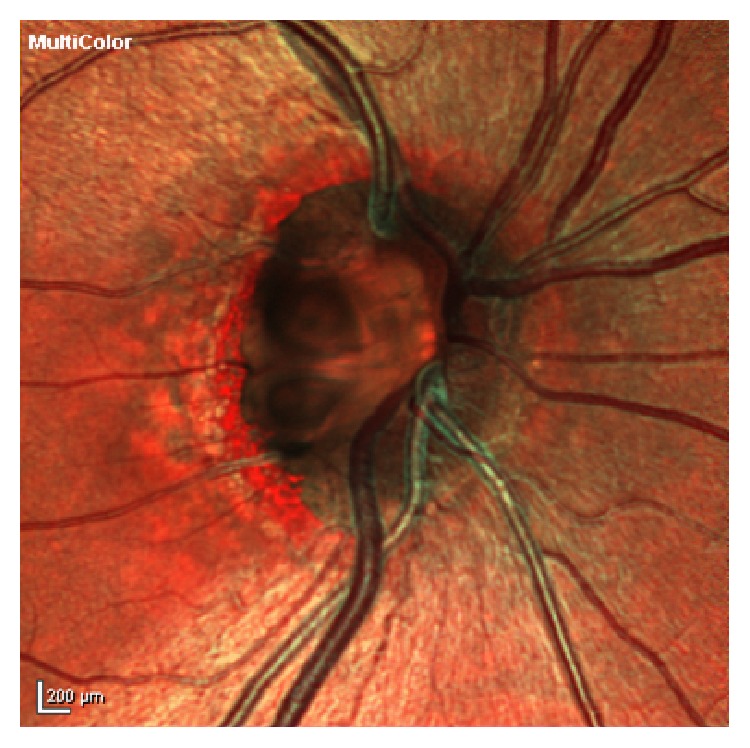
High definition fundal photo demonstrating architecture of double optic disc pit.

On examination he was found to have a Snellen's visual acuity of 6/18 in the right improving with pinhole to 6/12 and 6/5 in the left. Anterior segment examination was unremarkable. Intraocular pressure was 15 mmHg in the right and 16 mmHg in the left. Dilated fundoscopy revealed a double optic disc pit in the right eye (Figure 1). Retina and macula were flat; there was no fluid or detachment. The left optic disc was normal.

**Figure 2:**
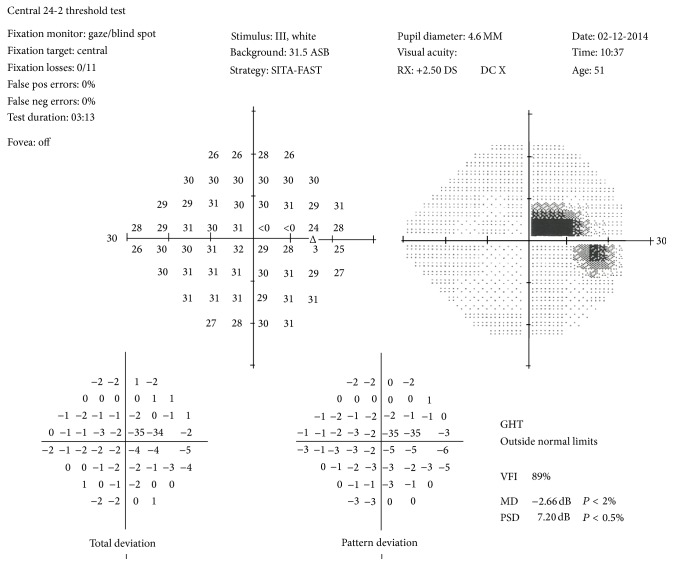
Paracentral scotoma in right visual field.

OCT of the macula and disc was otherwise unremarkable. Humphrey's visual field test revealed a paracentral scotoma in the right (Figure 2) and a normal left field. As he did not have any pit related maculopathy, he was able to be discharged with advice regarding signs of maculopathy. He will continue to attend his opticians every 2 years.

## 3. Discussion

Optic disc pits (ODP) were first described by Wiethe in 1882 [1, 2]. They are rare entities with an incidence of 1 in 11,000 and they occur equally in males and females [2]. 15% are bilateral [2]. They are seen as small depressions which tend to be located in the inferior temporal sector [1, 4]. Colours can vary, but they are most commonly grey [2]. The pathophysiology behind ODP remains unclear [2].

Only three previous cases noting double optic disc pits have been found on PubMed.

Investigations include OCT which demonstrates a bilaminar structure [5] and visual fields which may show arcuate scotomas or an enlarged blind spot [1, 2]. Although ODP position and field defects do not always correspond [2], it has been hypothesised previously that damage to the nerve fibre layer may cause the visual field defects [3].

Histology reveals dysplastic retina that herniates through the lamina cribrosa into a collagen rich area [1, 2]. It may extend further into the subarachnoid space [1, 2].

Patients often remain asymptomatic until they develop optic disc maculopathy [3]. Interestingly in our case though the patient had visual disturbance; he did not have any signs of maculopathy. His visual reduction could be accounted for by potential damage to the nerve fibre layer by the ODP [3].

Serous maculopathy occurs in 25–75% of cases and is more common in men [2, 6]. These patients usually present in their third or fourth decade of life [1, 2].

Treatment of maculopathy includes standalone laser or vitrectomy with or without laser [1, 2, 5]. If left untreated, the visual prognosis is poor [1, 2, 5].

Optic disc pits are an unusual entity, and double optic disc pits are extremely rare. Although patients are usually asymptomatic; they may develop symptoms due to maculopathy. Prompt identification and treatment of complications are required to prevent a poor visual prognosis.
